# The synergistic antitumor activity of 3-(2-nitrophenyl) propionic acid-paclitaxel nanoparticles (NPPA-PTX NPs) and anti-PD-L1 antibody inducing immunogenic cell death

**DOI:** 10.1080/10717544.2021.1909180

**Published:** 2021-04-19

**Authors:** Xiao-Chuan Duan, Li-Yuan Peng, Xin Yao, Mei-Qi Xu, Hui Li, Shuai-Qiang Zhang, Zhuo-Yue Li, Jing-Ru Wang, Zhen-Han Feng, Guang-Xue Wang, Ai Liao, Ying Chen, Xuan Zhang

**Affiliations:** a Beijing Key Laboratory of Molecular Pharmaceutics and New Drug Delivery Systems, School of Pharmaceutical Sciences, Peking University, Beijing, People’s Republic of China; b Department of Pharmaceutics, School of Pharmaceutical Sciences, Peking University, Beijing, People’s Republic of China; c Tianjin Key Laboratory on Technologies Enabling Development Clinical Therapeutics and Diagnostics (Theranostics), School of Pharmacy, Tianjin Medical University, Tianjin, People’s Republic of China

**Keywords:** 3-(2-nitrophenyl) propionic acid-paclitaxel, nanoparticles, immunogenic cell death, anti-PD-L1 antibody, immune response, synergistic antitumor activity

## Abstract

Cancer immunotherapy is a strategy that is moving to the frontier of cancer treatment in the current decade. In this study, we show evidence that 3-(2-nitrophenyl) propionic acid-paclitaxel nanoparticles (NPPA-PTX NPs), act as immunogenic cell death (ICD) inducers, stimulating an antitumor response which results in synergistic antitumor activity by combining anti-PD-L1 antibody (aPD-L1) *in vivo*. To investigate the antitumor immunity induced by NPPA-PTX NPs, the expression of both ICD marker calreticulin (CRT) and high mobility group box 1 (HMGB1) were analyzed. In addition, the antitumor activity of NPPA-PTX NPs combined with aPD-L1 *in vivo* was also investigated. The immune response was also measured through quantitation of the infiltration of T cells and the secretion of pro-inflammatory cytokines. The results demonstrate that NPPA-PTX NPs induce ICD of MDA-MB-231 and 4T1 cells through upregulation of CRT and HMGB1, reactivating the antitumor immunity via recruitment of infiltrating CD3+, CD4+, CD8+ T cells, secreting IFN-γ, TNF-α, and the enhanced antitumor activity by combining with aPD-L1. These data suggest that the combined therapy has a synergistic antitumor activity and has the potential to be developed into a novel therapeutic regimen for cancer patients.

## 1. Introduction

Cancer immunotherapy is a therapeutic strategy that stimulates the body’s immune system against tumors, it is quickly becoming the future of cancer treatment in modern times (Couzin-Frankel, 2013). Up to now, the best and most successful strategy is to block the checkpoint molecules which suppress the antitumor immunity to reactivate the immune system, resulting in the inhibition of tumor growth. Immune checkpoint blockade (ICB) therapy using monoclonal antibodies to block the programmed cell death protein 1/programmed cell death ligand 1 (PD-1/PD-L1) axis has emerged as a promising approach for clinical treatments of cancers such as bladder cancer and non-small cell lung cancer (Powles et al., 2014; Peters et al., 2017; Rittmeyer et al., 2017). Immune checkpoint inhibitors release the immune suppression caused by tumorigenesis (Dunn et al., 2002; Vinay et al., 2015), thereby reactivating the T cells, leading to the rediscovery and eradication of tumor cells by the host immune system.

Chemotherapy-induced ICD of tumors and the immunogenic dead tumor cells can release tumor-associated antigens (TAA) and damage-associated molecular patterns (DAMPs), including calreticulin (CRT) and high mobility group box 1 (HMGB1) (Kepp et al., 2011; Galluzzi et al., 2017; Feng et al., 2018; Wang Q et al., 2018). Specifically, the endoplasmic reticulum chaperone protein, CRT, translocations to the tumor cell surface and generates an essential ‘eat-me’ signal for dendritic cell (DCs) engulfment and tumor antigen uptake (Obeid et al., 2007; Schiavoni et al., 2011). The secretion of HMGB1 from the nucleus to the cytoplasm is followed by HMGB1 release into the extracellular matrix of dying tumor cells. The secreted HMGB1 interacts with TLR4 expressed on DCs, thereby stimulating antigen presentation by DCs as well as DC production of IL1β, which activates CD8+ T cells (Apetoh et al., 2007; Zitvogel et al., 2008). The most-well-known ICD inducers include doxorubicin, mitoxantrone, oxaliplatin (OXP), bortezomib, and cyclophosphamide (Bezu et al., 2015). When combined with immunotherapy, the antitumor effect and duration of immune checkpoint blockade therapy have significantly improved due to the TAA released from the tumor cells. The TAA release triggers host production of durable and effective tumor antigen-specific T cells and synergistically optimizes the antitumor effects (Emens & Middleton, 2015; Yoshinari et al., 2015). In addition, chemotherapy enhanced immunosuppression caused by the overexpression of PD-L1 can be abolished when incorporating ICB-based immunotherapy into the treatment strategy, through which a synergistic anticancer activity can be expected (Peng et al., 2015; Wang C et al., 2018).

Chemotherapy is regarded as a proper candidate for combination with ICB therapy in pre-clinical studies or even in clinical trials (Rizvi et al., 2016; Bellmunt et al., 2017; Weiss et al., 2017; Mei et al., 2018). PTX is an effective antitumor chemotherapeutic agent with wide clinical use (Meng et al., 2016). There is growing evidence indicating that PTX can induce ICD and augment antitumor immunity (Kim et al., 2020; Lau et al., 2020; Su et al., 2020; Tu et al., 2020; Yang et al., 2020; Wang et al., 2021). The phase 3 study, IMpassion130, evaluated atezolizumab, a monoclonal antibody targeting PD-L1, plus nab-paclitaxel compared with placebo plus nab-paclitaxel as a first-line treatment for patients with unresectable locally advanced or metastatic triple-negative breast cancer (Schmid et al., 2018), which was approved by the U.S. FDA in 2019.

The prodrug nanomedicine based on the molecular self-assembly of amphiphilic prodrugs was developed in decades due to their higher loading efficiency and enhanced therapeutic efficiency (Li et al., 2019). In previous research (Song et al., 2016; Duan et al., 2019), PTX was conjugated with 3-(2-nitrophenyl) propionic acid (NPPA) to obtain a novel bioreductive PTX prodrug (NPPA-PTX). The results indicated that NPPA-PTX could completely convert to active PTX in tumor tissues and produce anti-tumor activity. Furthermore, NPPA-PTX could self-assemble to form nanoparticles (NPs). The safety and antitumor activity of NPPA-PTX NPs were confirmed through both *in vitro* and *in vivo* experiments.

In the present study, the bioreductive PTX prodrug NPPA-PTX was synthesized and formulated NPPA-PTX NPs. The evidence of NPPA-PTX NPs acting as an ICD inducer *in vitro* is reported. Pharmacokinetics and biodistribution of NPPA-PTX NPs were also investigated. The antitumor activity and immune response of NPPA-PTX NPs combined with anti-PD-L1 antibody (aPD-L1) *in vivo* were also studied. We suggest that the combined therapy show synergistic antitumor activity and have the potential to be developed into a novel therapeutic regime for cancer patients.

## 2. Materials and methods

### 2.1. Materials

#### 2.1.1. Drugs and antibodies

NPPA was purchased from Accela ChemBio Co. Ltd (Shanghai, China). PTX, OXP, and cisplatin (CDDP) were purchased from Heowns OPDE Technologies, LLC (Tianjin, China). NPPA-PTX was synthesized from NPPA and PTX as previously published (Song et al., 2016). 6-FAM azide and Cy7-NHS ester were obtained from AAT Bioquest, Inc. (Sunnyvale, CA, USA). DSPE-PEG_2000_-NH_2_ and DSPE-mPEG_2000_ were obtained from Nanosoft Bitechnology LLC (Winston-Salem, NC, USA). Sulforhodamine B (SRB) was acquired from Macklin Biochemical Co., Ltd (Shanghai, China). EIPA and Filipin complex was provided by MedChemExpres LLC (Monmouth Junction, NJ, USA). Methyl-β-cyclodextrin, chlorpromazine hydrochloride, and D-(+)-Sucrose were purchased from J&K Scientific Ltd. (Beijing, China). Taxol^®^ was purchased from Cisen Pharmaceutical Co., Ltd. (Shandong, China). RIPA lysis buffer containing protease inhibitor was obtained from Solarbio (Beijing, China). BCA protein assay kits were acquired from Invitrogen, ThermoFisher Scientific Inc. (Waltham, MA, USA).

Rab5 (C8B1) Rabbit mAb (#3547), Rab7 (D95F2) XP^®^ Rabbit mAb (#9367), LAMP1 (D2D11) XP^®^ Rabbit mAb (#9091), GM130 (D6B1) XP^®^ Rabbit mAb (#12480), Calreticulin (D3E6) XP^®^ Rabbit mAb (#12238), Alexa Fluor^®^ 647 Conjugate (#4414), CD3ε (D7A6E™) XP^®^ Rabbit mAb (#85061), CD4 (D7D2Z) Rabbit mAb (#25229), CD8α (D4W2Z) XP^®^ Rabbit mAb (Mouse Specific) (#98941), PD-L1 (D5V3B) Rabbit mAb (Mouse Specific; IHC Specific) (#64988), β-Actin (13E5) Rabbit mAb (#4970) and Anti-rabbit IgG, HRP-linked Antibody (#7074) were purchased from Cell Signaling Technology, Inc. (Danvers, MA, USA). Golgi-tracker (C1043), ER-tracker (C1041), and Hoechst 33342 (C1028) were purchased from Beyotime Biotechnology Inc. (Shanghai, China). Anti-HMGB1 (ab79823) and anti-FOXP3 (ab215206) were purchased from Abcam (Cambridge, MA, USA). InVivoMAb anti-mouse PD-L1 (B7-H1) (BE0101) was provided by Bio X Cell (Lebanon, NH, USA). Cell culture media L-15, McCoy’s 5 A, DMEM, RPMI1640, penicillin, streptomycin, FBS, and L-glutamine were all obtained from Thermo Fisher Scientific (Waltham, MA, USA).

#### 2.1.2. Cell culture

The human cell lines including mammary carcinoma (MDA-MB-231) and colorectal carcinoma (HCT116) were purchased from American Type Culture Collection (ATCC, Rockefeller, MD, USA) and cultivated in L-15 and McCoy’s 5 A medium respectively. The mouse mammary carcinoma (4T1) and colon carcinoma (CT26) were obtained from the Chinese Academy of Sciences Cells Bank (Shanghai, China) and were cultivated in DMEM (high glucose) and RPMI1640 medium. All cell culture medium was supplemented with 10% FBS, 100 units mL^−1^ penicillin, and 100 μg mL^−1^ streptomycin. The cultures were maintained at 37 °C, 95% relative humidity.

#### 2.1.3. Animals

Female BALB/c nude mice (20 ± 2 g), female BALB/c mice (20 ± 2 g), and male SD rats (200 ± 20 g) (all supplied by SPF (Beijing) Biotechnology Co., Ltd., Beijing, China) were acclimatized for 7 days prior to the experiment and allowed free access to a standard diet and water with the maintained condition of 25 °C temperature and 50% relative humidity. All care and handling of animals were performed with the approval of the Experimental Animal Center of Peking University Health Science Center. Additionally, this study was performed following the National Institutes of Health guidelines for the use of experimental animals.

To prepare the MDA-MB-231 or HCT116 tumor-bearing nude mice, the female BALB/c nude mice were injected subcutaneously with 0.2 mL MDA-MB-231 or HCT116 cell suspension (1 × 10^7^ cells). 4T1 or CT26 tumor-bearing mice were prepared by injecting subcutaneously with 0.2 mL 4T1 or CT26 cell suspension (1 × 10^6^ cells) into the right flank of BALB/c mice.

### 2.2. Preparation and characterization of NPPA-PTX nanoparticles

The synthesis of NPPA-PTX (Supplementary Scheme 1) and the preparation of NPPA-PTX NPs were reported in our previous work (Song et al., 2016; Duan et al., 2019). NPPA-PTX-FAM was synthesized through click reaction by alkynylation modified NPPA-PTX and 6-FAM-Azid (Supplementary Schemes 2 and 3), and NPPA-PTX-FAM NPs were prepared by the same formulation as the previous research (Duan et al., 2019).

Particle size and zeta potential were determined by dynamic light scattering (DLS) measurements (Malvern Zetasizer Nano ZS90, Malvern, UK). The morphology was examined by transmission electron microscopy (TEM) on the HT7700 microscope (HITACHI, Japan). For surface chemical properties characterization, X-ray photoelectron spectroscopy (XPS, Axis Ultra DLD, Kratos Analytical Ltd., Germany) and the H-NMR (AVANCE III 400 MHz, Bruker, Switzerland) were examined. For H-NMR, NPPA-PTX NPs were prepared by D_2_O instead. The dilution stability of NPPA-PTX NPs was measure by DLS under different dilution ratios by deionized water. Drug loading capacity (DLC) was calculated as DLC% = w (NPPA-PTX)/w (NPPA-PTX NPs).

The release of NPPA-PTX from NPPA-PTX NPs was investigated by the dialysis method. Briefly, a sample of NPPA-PTX NPs (1 mL, 0.1 mg mL^−1^) was placed in a dialysis tube (MWCO 7000) and tightly sealed. Then, the dialysis tube was immersed in 40 mL 10 mM PBS (pH 7.4) with a 0.5% tween 80 to meet the sink condition. Taxol was prepared by the same method as a control. The dialysis tubes were incubated in an orbital shaker at 37 °C. Samples (0.2 mL) were collected at a predetermined time from the release medium over a period of 96 h, and the same volume of fresh medium was refilled to the release medium after every sample collection. The concentration of NPPA-PTX and PTX was determined by HPLC.

### 2.3. Endocytosis mechanism and intracellular fate of NPPA-PTX NPs

MDA-MB-231 or HCT116 cells were seeded in a 6-well flat-bottom tissue-culture plate at a destiny of 3 × 10^5^ cells well^−1^ with 3 mL growth medium. After 24 h, the medium was replaced with NPPA-PTX NPs medium solution (10 μM) and incubated for 2, 4, or 6 h at 37 °C. After incubation, the cellular uptake of drugs was measured by HPLC analysis. The endocytosis mechanism was investigated via a pharmacological inhibition strategy. After cells were incubated with various inhibitors (listed in Table S1) for 60 min at 37 °C, the medium was removed, and NPPA-PTX NPs medium solution (10 μM) (containing the same concentration of inhibitors) was added for another 5 h of incubation. The amount of drug endocytosed was determined by HPLC.

**Table 1.** The main pharmacokinetic parameters of NPPA-PTX, released PTX from NPPA-PTX, and PTX from Taxol after intravenous administration of the NPPA-PTX NPs (6.04 mg kg^−1^, equimolar with 5.00 mg kg^−1^ PTX) and Taxol (5.00 mg kg^−1^) in rats.

Subject	*T*_max_ (h)	*C*_max_ (ng mL^−1^)	AUC_last_ (h ng mL^−1^)	AUCINF_obs (h ng mL^−1^)	Vz_obs (mL kg^−1^)	Cl_obs (mL h^−1^ kg^−1^)	MRTlast (h)	MRTINF_obs (h)	HL_Lambda_z (h)
NPPA-PTX	0.0333 ± 0	4580 ± 2140	3810 ± 2640	3930 ± 2710	20,700 ± 9320	1990 ± 987	2.91 ± 0.280	3.90 ± 0.656	7.45 ± 0.915
PTX released from NPPA-PTX	0.0333 ± 0	99.0 ± 29.2	695 ± 99.6	837 ± 264**	93,700 ± 26,400**	7650 ± 2050	7.38 ± 0.817	12.4 ± 6.36*	9.53 ± 5.82
PTX from Taxol	0.0333 ± 0	8600 ± 2500	4480 ± 1310	4560 ± 1310	10,300 ± 5480	1170 ± 398	2.50 ± 0.545	3.04 ± 0.791	5.86 ± 1.24

*Note*. Values are shown as mean ± SEM, *n* = 3.

*p < .05, **p < .01, compared with PTX from Taxol.

Confocal fluorescence microscope was used to observe the fluorescence distribution of NPPA-PTX-FAM NPs in MDA-MB-231 or HCT116 cells. The cells were plated in confocal dishes (20 mm) at the density of 1 × 10^5^ cells per well for 24 h. Then NPPA-PTX-FAM NPs were exposed to cells and incubated for 2 h at 37 °C. Then the cells were washed and stained with cell trackers (ER-tracker, Golgi-tracker) or co-incubated with at optimal antibody concentration (anti-Rab5 (1:200), anti-Rab7 (1:200), and anti-LAMP1 (1:200)) as defined by the manufacturer protocol. The cells were further stained with secondary antibody followed by the staining of Hoechst 33342. After that, samples were observed by a CLSM immediately (Olympus FV1000, Olympus Corporation, Tokyo, Japan).

### 2.4. The expression of CRT and HMGB1 on tumor cells

The expression of CRT on the surface of the MDA-MB-231 or 4T1 cells was determined by immunofluorescence measurement. Briefly, cells were plated in confocal dishes (20 mm) at the density of 5 × 10^4^ cells per well for 24 h. Then cells were incubated with NPPA-PTX NPs (10 μM) for 12 h, and CDDP (150 μM), OXP (300 μM) for 4 h at 37 °C. The cells were washed twice with PBS and fixed with 4% paraformaldehyde at room temperature for 20 min, blocked with BSA solution for 30 min, and then incubated with anti-CRT antibody overnight at 4 °C. The cells were then washed twice with PBS and incubated at room temperature for 1 h with Alexa fluor 647-conjugated secondary antibody. The cell samples were observed under a CLSM. The data was analyzed based on fluorescence and laser scattering intensities by FV10-ASW 4.2 software.

To detect the HMGB1 secretion, MDA-MB-231 or 4T1 cells were seeded in a 6 cm cell culture dish at the density of 3.5 × 10^5^ cells and cultured for 24 h. Then the cells were treated with NPPA-PTX NPs (10 μM) for 12 h, and CDDP (150 μM), OXP (300 μM), for 4 h at 37 °C. For the extraction of whole proteins, the cells were washed twice with pre-cold phosphate-buffered saline (PBS), and then treated with RIPA lysis buffer containing 1% protease inhibitor PMSF to obtain cell lysates. The lysates were sonicated and centrifuged at 4 °C for 15 min at a centrifugal force of 12,000 *g*. The protein in the supernatants was quantified using the BCA protein assay kit, and then stored at −20 °C. Cell lysates containing equal amounts of protein (30 mg protein per lane) were loaded on a 12% sodium dodecyl sulfate-polyacrylamide gel and electrophoresed at 80 mV for 30 min and 100 mV for 2 h. Then the proteins were transferred to Polyvinylidene Fluoride (PVDF) membranes at 110 mA for 2 h, and then blocked for 2 h in a blocking solution with 5% skim milk powder in Tris-Buffered Saline Tween-20 (TBST) buffer for 1 h at room temperature. The membranes were then incubated with primary antibodies against HMGB1 (1:10,000 dilution) or β-actin (1:2000 dilution) in 5% skim milk overnight at 4 °C with shaking. Membranes were then incubated with secondary antibodies (1:2000 dilution ratio) conjugated with horseradish peroxidase for 1 h, and washed with TBST three times. The bands were visualized using enhanced chemiluminescence on a UVITEC NineAlliance Q9 system (UVITEC Ltd., Cambridge, UK) and grayscale analysis was performed using ImageJ 1.51j8 software (National Institutes of Health, Bethesda, MD, USA).

### 2.5. Pharmacokinetics and biodistribution of NPPA-PTX NPs

To determine the drug blood retention effect of NPPA-PTX NPs, the plasma pharmacokinetics (PK) of NPPA-PTX and released PTX from NPPA-PTX was quantified. Briefly, 6 male SD rats (200 ± 20 g) were randomly divided into two groups (*n* = 3) and intravenously administered with Taxol (5.00 mg kg^−1^) and NPPA-PTX NPs (6.04 mg kg^−1^, equimolar to 5.00 mg kg^−1^ PTX), respectively, via tail vein. Post injection, ∼200 μL blood samples were drawn from the jugular vein at predetermined time points of 0.033, 0.083, 0.25, 0.5, 1, 2, 4, 6, 8, 12, and 24 h post-injection and centrifuged immediately at 3000 rpm for 10 min at 4 °C. Then 40 μL plasma samples were collected and 150 μL of acetonitrile was added followed by centrifugation at 12,000 rpm for 10 min to extract the NPPA-PTX, released PTX from NPPA-PTX and PTX from Taxol. 200 μL of deionized water was added into the 40 μL supernatants followed by vortexing for 1 min. Finally, the pharmacokinetic data analysis was performed via a liquid chromatography-tandem mass spectrometry (LC-MS/MS Applied Biosystems. Foster City, CA, USA) and pharmacokinetic parameters were acquired by Winnolin 7.0 software.

Meanwhile, MDA-MB-231 tumor-bearing mice were randomly grouped (*n* = 3) when the tumor volume reached ∼400 mm^3^. Then each group was intravenously injected with Taxol (10.00 mg kg^−1^) and NPPA-PTX NPs (12.10 mg kg^−1^, equimolar to 10.00 mg kg^−1^ PTX) via tail vein. Mice were sacrificed and the tumors and main organs were harvested at 1, 4, 8, and 24 h after administration. Each tissue sample was homogenized in 1 mL acetonitrile and centrifugated. The concentrations of NPPA-PTX, released PTX from NPPA-PTX, and PTX from Taxol in the extract were determined by LC-MS/MS.

The *in vivo* imaging of the NPPA-PTX NPs was evaluated in HCT116 tumor-bearing nude mice by using Cy7 labeled NPPA-PTX NPs on an IVIS Spectrum (PerkinElmer Inc., Waltham, MA, USA). Mice were sacrificed and the tumor tissues were obtained at 24 h.

### 2.6. Antitumor activity of NPPA-PTX NPs in vitro and in vivo

The *in vitro* cytotoxicity of NPPA-PTX NPs against MDA-MB-231 or HCT116 cell lines was measured by the SRB method and the absorbance at 540 nm was examined with a 96-well plate reader (BioTek Synergy HTX, BioTek Instruments, Inc., Winooski, VT, USA). Dose-effect curves were constructed and IC50 values were calculated by GraphPad Prism6/SPSS Software.

MDA-MB-231 or HCT116 tumor-bearing nude mice were randomly grouped (*n* = 6) when tumor volume reached ∼100 mm^3^. Each group was intravenously administered with physiological saline, Taxol (10 mg kg^−1^, q3d × 4), NPPA-PTX NPs (12 mg/kg^−1^, equimolar to 10 mg kg^−1^ PTX, q3d × 4), and NPPA-PTX NPs (36 mg kg^−1^, equimolar to 10 mg kg^−1^ PTX, q3d × 4), respectively. The tumor volume and body weight was measured every 2 days. After 17 days all mice were sacrificed. Tumor tissues were isolated and photographed. Other organs (heart, liver, spleen, lung, and kidney) were isolated for H&E staining, blood samples were collected, and the hematological and biochemical parameters were examined.

### 2.7. Antitumor activity of NPPA-PTX NPs combined with aPD-L1

To evaluate the antitumor activity of NPPA-PTX NPs combined with aPD-L1, 4T1, or CT26 tumor-bearing mice were randomly grouped (*n* = 5) when the tumor volume reached 50 ∼ 100 mm^3^. Each group was intravenously administered with physiological saline, NPPA-PTX NPs (12 mg kg^−1^, q3d × 4), and NPPA-PTX NPs (12 mg kg^−1^, q3d × 4) combined with aPD-L1 (24 h postinjection, i.p. 100 μg per mouse), respectively. The tumor volume and body weight were measured every 2 days. The tumor volume was calculated based on the equation (*a* × *b*^2^)/2, where *a* and *b* are the length and width of the tumor, respectively. On day 15, all of the mice were sacrificed, and the tumor tissues were isolated and photographed. Tumors were then harvested for histological evaluation.

### 2.8. Immunohistochemical (IHC) staining

Tumors were harvested, fixed in formalin, and embedded in paraffin before being cut into 4 μm sections. Sections for IHC were deparaffinized, rehydrated, and subjected to antigen retrieval for 30 min in 98 °C 10 mM sodium citrate buffer (pH 6.0). After antigen retrieval, slides were blocked in PBS (pH 7.4) supplemented with 10% BSA and 0.5% goat serum prior to incubation with primary antibodies overnight at 4 °C. Staining was visualized by 3,3′-diaminebenzidine (DAB) system. Slides were washed and incubated with endogenous horseradish peroxidase blocker for 10 min and goat anti-rabbit IgG-HRP (ZSGB-Bio, Beijing, China) for 20 min, respectively. A positive reaction was detected by exposure to DAB according to the manufacturer’s instructions. Slides were counterstained with hematoxylin and visualized under a bright field microscope at 200 × or 400 × magnification.

### 2.9. ELISA

Mice were sacrificed at the end of the experiments, tumors and serum were collected. An equal amount of tumor tissues were taken and suspended in an equal volume of PBS, homogenized with a homogenizer, then centrifuged at 12,000 rpm for 10 min. The supernatant was collected for detecting the levels of TNF-α and IFN-γ according to the manufacturer’s instructions (CUSABIO TECHNOLOGY LLC, Houston, TX, USA). The cytokine levels in serum were also measured.

### 2.10. Statistical analysis

All experiment was conducted in triplicate and the data were presented as mean ± standard deviation (SD). Statistical tests included unpaired one-tailed and two-tailed Student’s *t*-tests using Welch’s correction and one-way ANOVA followed by multiple comparison tests. Statistical significance was defined as *p* < .05.

## 3. Results

### 3.1. Characterization of NPPA-PTX NPs

**Figure 1.**
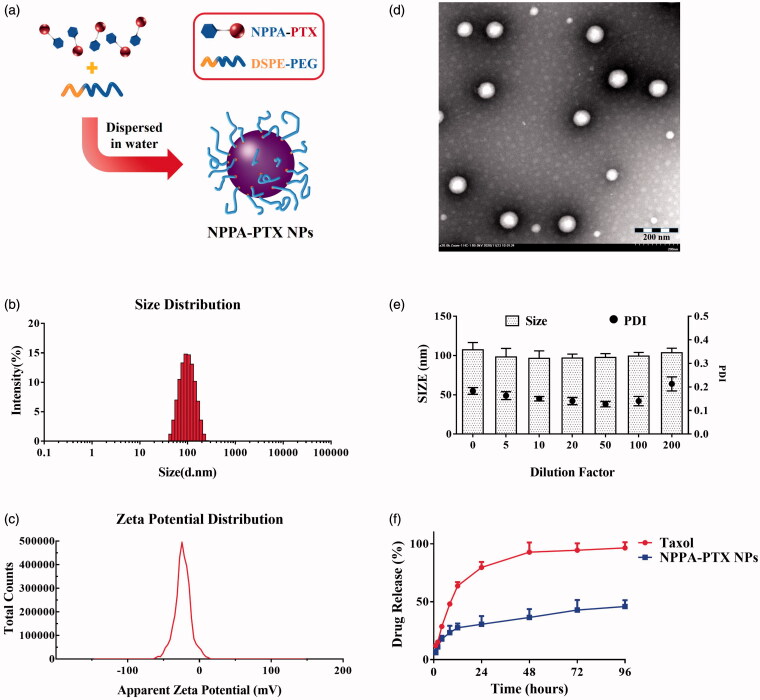
The preparation and characteristics of NPPA-PTX NPs. (a) NPPA-PTX NPs was prepared by precipitation. The mixed solution of NPPA-PTX and DSPE-PEG_2000_ (1:0.1, w/w) was dropped into deionized water while stirring, resulting in NPPA-PTX NPs occurring spontaneously. (b, c) Particle size and zeta potential of NPPA-PTX NPs. Particle size and zeta potential were determined by dynamic light scattering (DLS) measurements on Malvern Zetasizer Nano ZS90 instrument. The data were the mean of three tests. (d) The TEM image of NPPA-PTX NPs. NPPA-PTX NPs were carefully dropped onto a carbon-coated copper grid and then dried at room temperature overnight prior to observe on HT7700 microscope. (e) The dilution stability of NPPA-PTX NPs. The dilution stability of NPPA-PTX NPs was measure by DLS under different dilution ratios by deionized water. (f) *In vitro* drug release profiles of NPPA-PTX NPs and Taxol in 10 mM PBS (pH 7.4) with 0.5% tween 80 at 37 °C (*n* = 3, mean ± SEM was shown).

NPPA-PTX NPs were prepared by precipitation as shown in Figure 1(a). The size of NPPA-PTX NPs was 90.93 ± 0.75 nm (Figure 1(b)) and the polydispersity index was 0.115 ± 0.006. The zeta potential of NPPA-PTX NPs was −22.7 ± 2.3 mV (Figure 1(c)). The morphology of NPPA-PTX NPs was observed using TEM, showing a uniform spherical shape with a size of about 80 nm (Figure 1(d)). As seen in Figure 1(e), NPPA-PTX NPs could remain a stable particle size of about 90 nm even after a 200-fold dilution by water, which simulated blood dilution factors after intravenous injection *in vivo*. The DLC% of NPPA-PTX NPs was 86.3 ± 3.2%. The release rate of NPPA-PTX NPs and Taxol was shown in Figure 1(f). Compared with Taxol, NPPA-PTX NPs released NPPA-PTX in a controlled manner, the cumulative release of NPPA-PTX NPs and Taxol was 45.8 ± 5.5% and 96.5 ± 4.9% within 96 h, respectively.

To investigate the chemical composition of the nanoparticle surface, XPS was conducted on the NPPA-PTX NPs. The C1s XPS spectrum is shown in Figure S3(b). The binding peaks at energy values 284.76 eV and 286.27 eV are regarded as an indicator of –C–C– (55.10%) and –C–O– (35.29%) which are a portion of PEG. In Figure S3(c), the presence of PEG on the particle surface can be confirmed by an O–C peak (78.50%) at the binding energy 532.38 eV. Additionally, the peaks at binding energy 288.99 eV in C1s XPS spectrum and at the binding energy 533.59 eV in O1s XPS spectrum are indicators of –C=O– (8.11%) and –O=C–O– (21.50%), respectively, which indicate the presence of acetyl groups belonging to NPPA-PTX. Furthermore, the peaks at 2.5 ppm and 2.8 ppm in the ^1^H-NMR spectrum (Figure S3(e)) and their NOE signal in ^1^H–^1^H NOESY spectrum (Figure S3(f)) of NPPA-PTX NPs indicate the NOE effects between acetyl groups of different NPPA-PTX molecules. The peak at 3.6 ppm in Figure S3(e) is from the monomer (–(CH_2_–CH_2_–O)–) found in PEG. These data together demonstrate that NPPA-PTX NPs are covered by hydrophilic groups of PEG and acetyl groups of NPPA-PTX molecules on the surface.

### 3.2. Endocytosis mechanism and intracellular fate of NPPA-PTX NPs

**Figure 2.**
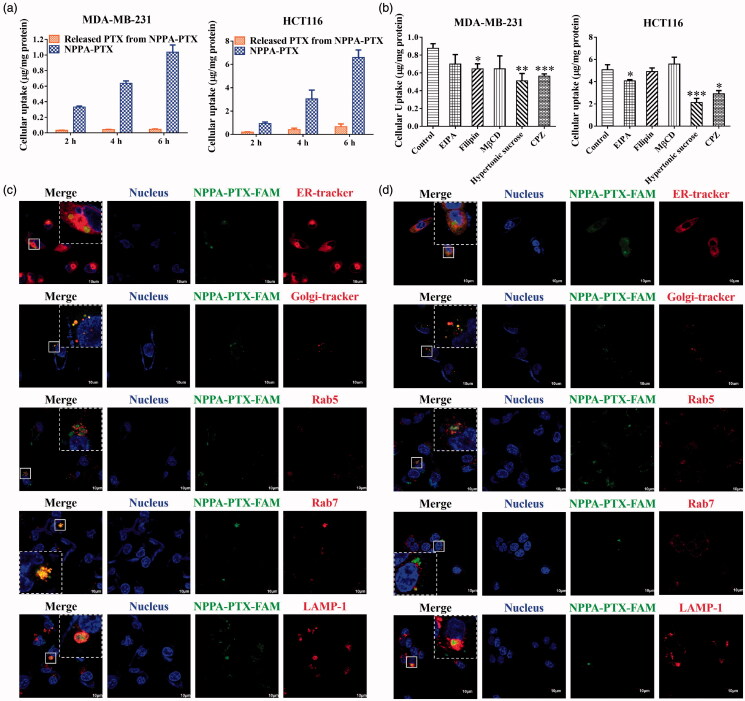
*In vitro* cellular uptake and endocytosis mechanism of NPPA-PTX NPs. (a) *In vitro* cellular uptake of NPPA-PTX NPs in MDA-MB-231 or HCT116 cells. (b) The endocytosis mechanism of NPPA-PTX NPs in MDA-MB-231 or HCT116 cells (*n* = 3, mean ± SEM was shown, *p < .05, **p < .01, ***p < .001). The intracellular transport pathways of NPPA-PTX-FAM NPs in MDA-MB-231 cells (c) or HCT116 cells (d).

As shown in Figure 2(a), the cellular uptake of NPPA-PTX NPs and released-PTX from NPPA-PTX increases with time in MDA-MB-231 or HCT116 cell lines. NPPA-PTX NPs exhibited different mechanisms of endocytosis in MDA-MB-231 or HCT116 cell lines (Figure 2(b)). The MDA-MB-231 cells, the inhibitions of filipin, chlorpromazine (CPZ), and hypertonic sucrose indicated that NPPA-PTX NPs were internalized through multiple pathways including caveolin-dependent endocytosis (CDE) and clathrin-mediated endocytosis (CME). In contrast, the endocytosis of NPPA-PTX NPs was inhibited by EIPA, CPZ, and hypertonic sucrose, which indicates that nanoparticles were internalized through CME and micropinocytosis in the HCT116 cells (He et al., 2013; Zhao & Stenzel, 2018).

To characterize the intracellular transport of NPPA-PTX NPs in MDA-MB-231 or HCT116 cell lines, NPPA-PTX-FAM NPs were established (Figure S4) and each pathway was analyzed via CLSM. As shown in Figure 2(c), part of the intracellular NPPA-PTX NPs was located in the ER and Golgi complex when it was internalized revealing that NPPA-PTX NPs involved in ER/Golgi route or secretion pathway. Part of nanoparticles colocalized with Rab5 (early endosomes), Rab7 (late endosomes) and LAMP1 (lysosomes), which indicates that NPPA-PTX NPs transports through the degradation pathway as well. In addition, the dispersed green signal outside the organelles indicates that NPPA-PTX NPs were also distributed in the cytoplasm. Similar results were observed in HCT116 cells (Figure 2(d)).

### 3.3. ICD Induced by NPPA-PTX NPs in vitro

**Figure 3.**
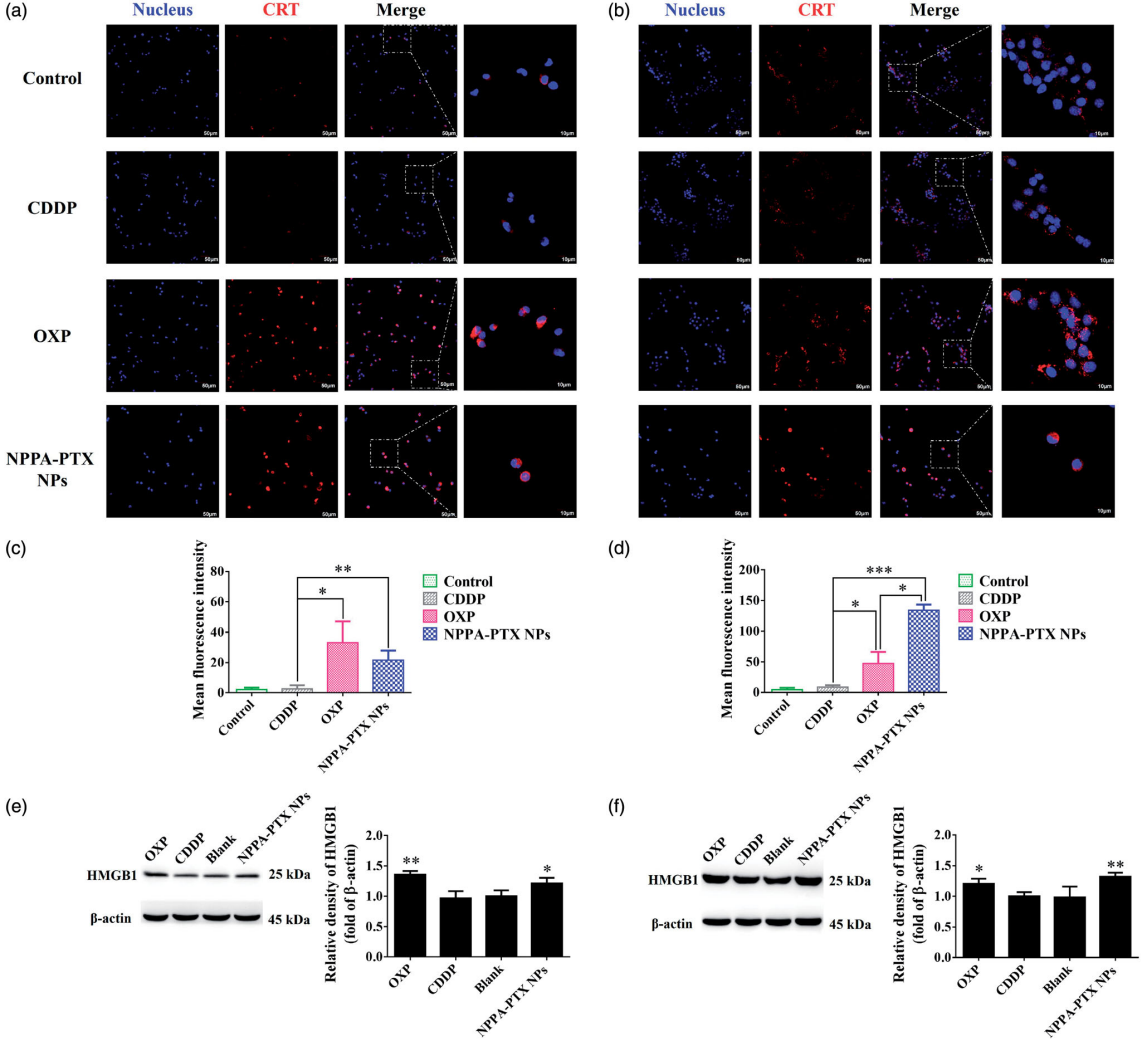
Immunogenic cell death with NPPA-PTX NPs *in vitro*. CLSM fluorescence images of calreticulin (CRT) expression on the surface of MDA-MB-231 cells (a) or 4T1 cells (b) treated with cisplatin (CDDP) (150 μM), oxaliplatin (OXP) (300 μM) and NPPA-PTX NPs (10 μM). Corresponding fluorescence intensity quantitative analysis of CRT expression on MDA-MB-231 cells (c) and 4T1 cells (d) (*n* = 3, mean ± SEM was shown, *p < .05, **p < .01, ***p < .001). Following the incubation with different drugs as above, the expression level of high mobility group box 1 (HMGB1) on MDA-MB-231 (e) or 4T1 (f) cells were determined by western blot and analyzed by ImageJ 1.51j8 software (*n* = 3, mean ± SEM was shown, *p < .05, **p < .01, compared with CDDP treatment group).

**Figure 4.**
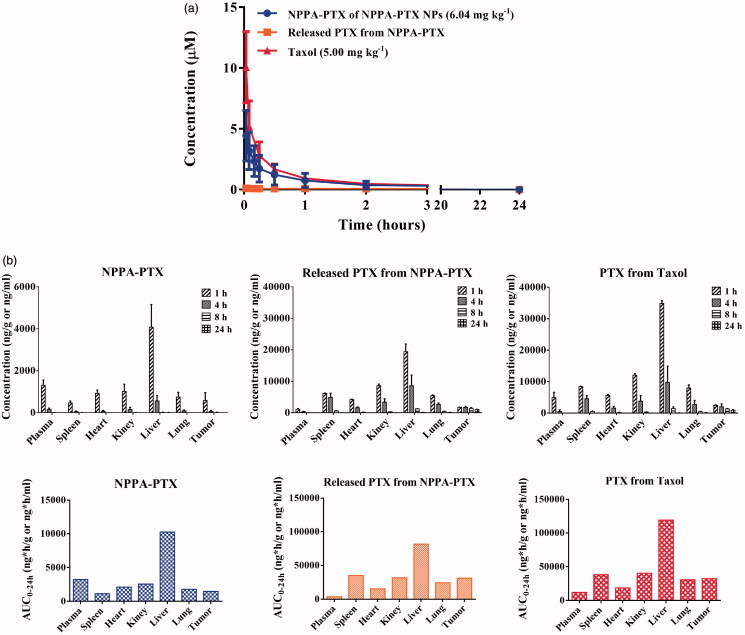
Pharmacokinetics and biodistribution assay of NPPA-PTX NPs and Taxol. (a) The plasma concentration–time curve of NPPA-PTX, released PTX from NPPA-PTX and PTX from Taxol after a single intravenous administration of NPPA-PTX NPs (6.04 mg kg^−1^, equimolar with 5.00 mg kg^−1^ PTX) and Taxol (5.00 mg kg^−1^) in SD rats. (b) The tissue distribution of NPPA-PTX, released PTX from NPPA-PTX and PTX from Taxol at 1, 4, 8, and 24 h after a single intravenous administration of NPPA-PTX NPs (12.10 mg kg^−1^, equimolar with 10.00 mg kg^−1^ PTX) and Taxol (10.00 mg kg^−1^) in MDA-MB-231 tumor-bearing nude mice.

The distinctive feature of ICD is the expression of CRT on the cell surface (Krysko et al., 2012; Sukkurwala et al., 2014). OXP, which induces ICD, was used as a positive control in this study (Tesniere et al., 2010). CDDP was used as a negative control (Martins et al., 2011). Surface CRT exposure on MDA-MB-231 and 4T1 cells were detected by immunofluorescence staining. The results suggested that OXP and NPPA-PTX NPs but not CDDP induce the translocation of CRT to the cell membrane in MDA-MB-231 (Figure 3(a)) and 4T1 (Figure 3(b)) cell lines. Quantitative analysis (Figure 3(c,d)) revealed that NPPA-PTX NPs reduced 35% percent of CRT expression in MDA-MB-231 cells than that of the OXP treatment group. 4T1 cells treated with NPPA-PTX NPs induced 2.8-fold higher CRT expression than that of the OXP treatment group (*p* < .05).

HMGB1 is another danger signal of ICD released from dying cells which can promote the production of pro-inflammatory cytokines and assist in proper antigen presentation (Sims et al., 2010). As confirmed by Western blotting analysis (Figure 3(e,f)), both OXP and NPPA-PTX NPs could significantly induce the HMGB1 expression, compared with CDDP treatment on MDA-MB-231 cells (*p* < .01, *p* < .05, respectively) or 4T1 cells (*p* < .05, *p* < .01, respectively). The HMGB1 ratio in NPPA-PTX NPs treatment group reduced protein expression by 11% percent in MDA-MB-231 cells than that of the OXP treatment group. Moreover, NPPA-PTX NPs induced a 1.1-fold higher expression of HMGB1 in 4T1 cells than that of the OXP treatment group.

### 3.4. Pharmacokinetics and biodistribution assay

The plasma concentration–time curve of NPPA-PTX, released PTX from NPPA-PTX, and PTX from Taxol were shown in Figure 4(a). The results observed that released PTX from NPPA-PTX was much lower than that of NPPA-PTX, indicating that NPPA-PTX NPs were stable in the circulating blood and distributed to the tissues as the intact NPPA-PTX NPs. The typical pharmacokinetic parameters of NPPA-PTX, released-PTX from NPPA-PTX, and PTX from Taxol were summarized in Table 1. The results determined that the AUC_0–∞_ of NPPA-PTX was 3930 (mg L^−1^)·h. Especially, the AUC_0–∞_ of released PTX from NPPA-PTX was 837 (mg L^−1^)·h, which was one-fifth of the AUC_0–∞_ (4560 (mg L^−1^)·h) of PTX in equivalent dosage of Taxol (*p* < .01). The apparent distribution volume (Vz) of NPPA-PTX and released PTX from NPPA-PTX was 20,700 mL kg^−1^ and 93,700 mL kg^−1^, respectively, while Vz of PTX from Taxol was 10,300 mL kg^−1^ in contrast (*p* < .01). Furthermore, the mean residence time (MRTINF) of NPPA-PTX and released PTX from NPPA-PTX was 3.90 h and 12.4 h, respectively. However, the mean residence time of PTX from Taxol was 3.04 h, which was about a quarter of that in released PTX from NPPA-PTX (*p* < .05).

The tissue distribution of NPPA-PTX NPs and Taxol are shown in (Figure 4(b)) The results revealed that NPPA-PTX NPs degraded rapidly to PTX in tumor tissue. The parameters are also summarized in Tables S2–S4. Compared with the AUC_0–24h_ of PTX from Taxol in plasma and tumor, the AUC_0–24h_ of released-PTX from NPPA-PTX showed lower values (about three tenths) in plasma but equivalent values in the tumor at the same time. Besides, comparing to PTX from Taxol, the ratio of AUC tissue/plasma of PTX released from NPPA-PTX is increased by 3.26 times.

The targeting effect of NPPA-PTX NPs was investigated in HCT116 tumor-bearing nude mice by using Cy7 labeled NPPA-PTX-Cy7 NPs. As shown in Figure S5, NPPA-PTX-Cy7 NPs have a stronger fluorescence signal in tumor than Cy7 dye injection used as control at all, the accumulation of fluorescent NPPA-PTX-Cy7 NPs existed in tumor site during the experiment duration (Figure S5(a)) and a higher fluorescence intensity was found in the NPPA-PTX-Cy7 NPs treatment group compared to that of Cy7 dye treatment group (Figure S5(b)).

**Figure 5.**
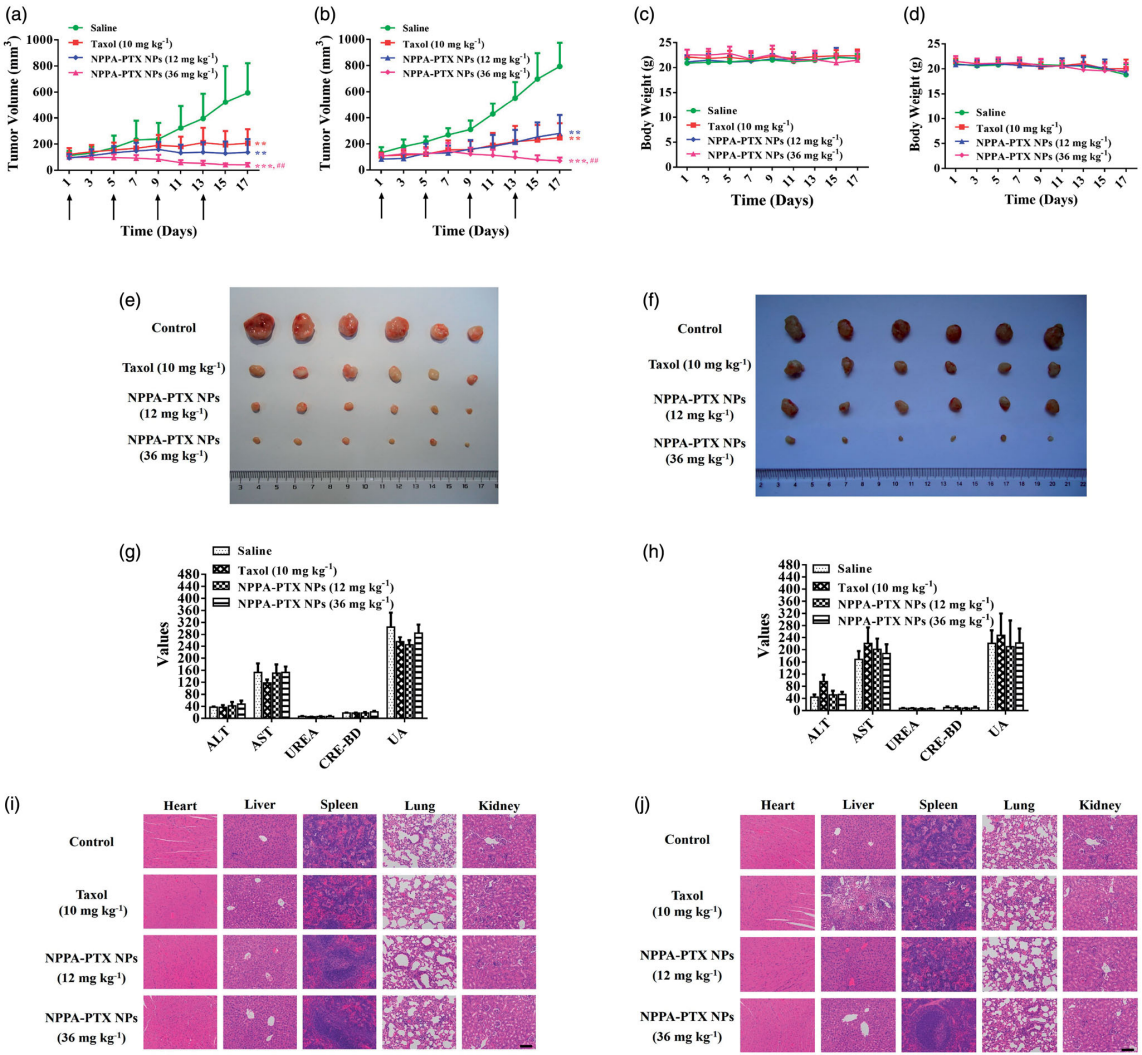
*In vivo* antitumor activity of NPPA-PTX NPs. MDA-MB-231 or HCT116 cells were implanted in the nude mice and randomly grouped when the tumor volume reached 100 ∼ 200 mm^3^. Then each group was intravenously administered with physiological saline, Taxol (10 mg kg^−1^, q3d × 4), NPPA-PTX NPs (12 mg kg^−1^, equimolar to 10 mg kg^−1^ PTX, q3d × 4) and NPPA-PTX NPs (36 mg kg^−1^, equimolar to 30 mg kg^−1^ PTX, q3d × 4) at day 1, respectively. Average tumor growth curves of each treatment group in MDA-MB-231 (a) or HCT116 (b) tumor-bearing nude mice. (*n* = 6, mean ± SEM was shown, **p < .01, ***p < .001 compared to the saline group; ^##^*p* < .01 compared to the Taxol group). Curves showing the body weight change of mice during various treatments in MDA-MB-231 (c) or HCT116 (d) tumor model. Tumor photograph from MDA-MB-231 (e) or HCT116 (f) tumor-bearing nude mice after treatment for 17 days. Levels of key indicators of liver and kidney functions in MDA-MB-231 (g) or HCT116 (h) tumor-bearing nude mice. H&E staining of main organs harvested from each treatment group in MDA-MB-231 (i) or HCT116 (j) tumor-bearing nude mice at experimental endpoint (scale bar = 100 μm).

### 3.5. Antitumor activity of NPPA-PTX NPs *in vitro* and *in vivo*

The *in vitro* cytotoxicity of NPPA-PTX NPs was evaluated in MDA-MB-231 or HCT116 cell lines and calculated IC50 values are shown in Table S5. The results indicate that the *in vitro* antitumor activity of NPPA-PTX NPs in MDA-MB-231 tumor cell line (0.072 ± 0.0045 μM) is significantly higher than that of PTX (0.063 ± 0.0033 μM, *p* < .05). But in HCT116 tumor cell line, the cytotoxicity of NPPA-PTX NPs (0.23 ± 0.02 μM) is significantly lower than that of PTX (0.30 ± 0.02 μM, *p* < .05).

The *in vivo* antitumor activity of NPPA-PTX NPs was evaluated in MDA-MB-231 and HCT116 tumor-bearing nude mice, respectively. As shown in Figure 5(a,b), tumor growth was significantly inhibited by Taxol (10 mg kg^−1^) and NPPA-PTX NPs treatment groups (12 mg kg^−1^; 36 mg kg^−1^) compared with the physiological saline treatment group (*p* < .01; *p* < .01; *p* < .001, respectively) in MDA-MB-231 or HCT116 tumor-bearing nude mice. Especially, NPPA-PTX NPs 36 mg kg^−1^ treatment group significantly inhibited the growth of MDA-MB-231 and HCT116 tumor compared with the Taxol treatment group (*p* < .01) in MDA-MB-231 or HCT116 tumor-bearing nude mice.

Drug safety evaluation in MDA-MB-231 or HCT116 tumor-bearing nude mice was also investigated. As shown in Figure 5(c,d), body weight was recorded every other day and no significant changes were perceived for all the groups throughout the tumor inhibition experiment in MDA-MB-231 and HCT116 tumor-bearing nude mice. In addition, biochemical analysis (Figure 5(g,h)) of the serum at the end of the antitumor studies in MDA-MB-231 and HCT116 tumor-bearing nude mice showed negligible damage on renal and hepatic functions. H&E staining results (Figure 5(i,j)) showed that there was no abnormal histology in major organs (i.e. heart, liver, spleen, lung, and kidney) upon NPPA-PTX NPs treatment groups at two dosages in MDA-MB-231 and HCT116 tumor-bearing nude mice.

### 3.6. Antitumor activity of NPPA-PTX NPs combined with aPD-L1 *in vivo*

**Figure 6.**
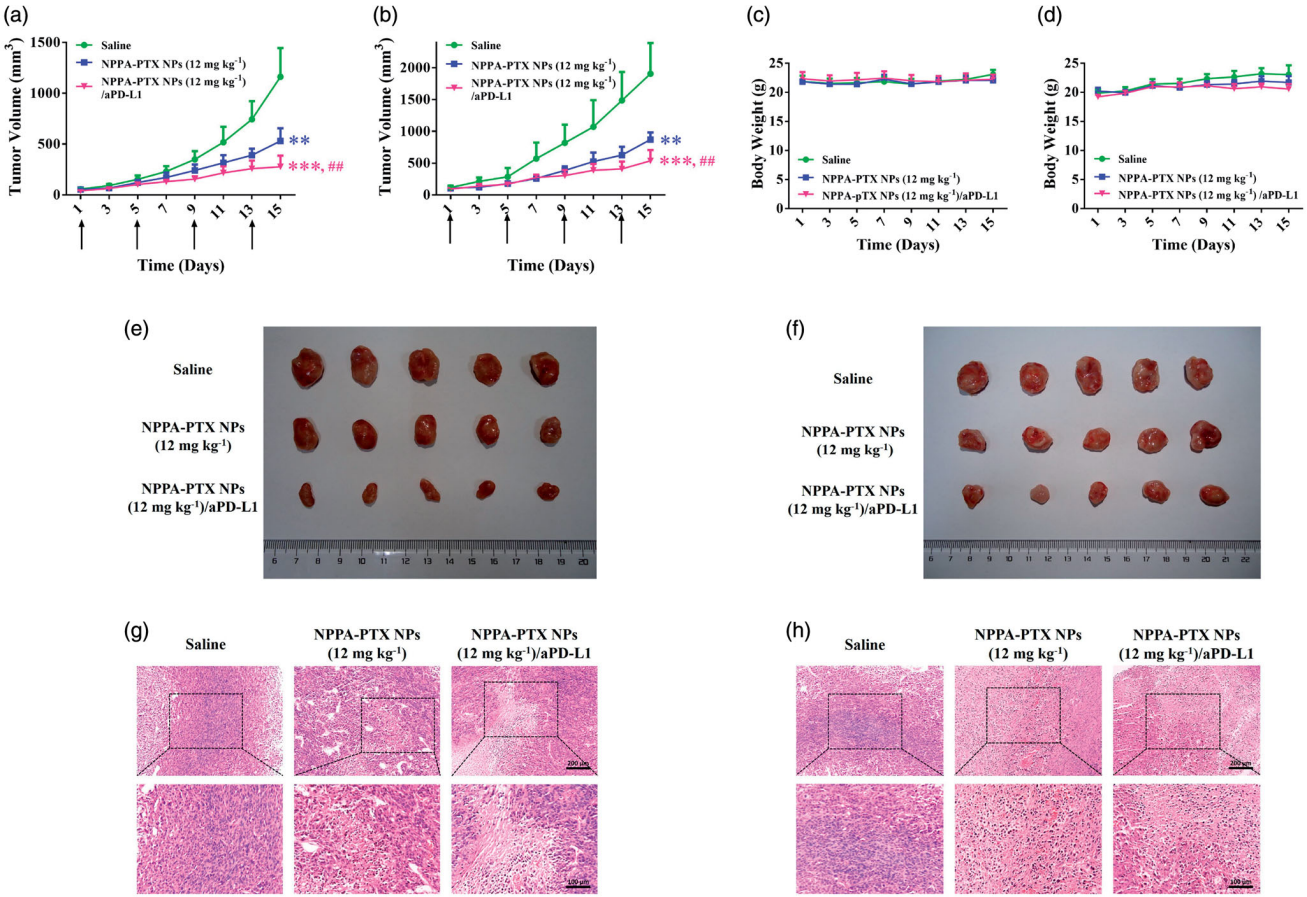
Antitumor activity of NPPA-PTX NPs combined with aPD-L1. 4T1 or CT26 cells were implanted in the mice and randomly grouped when the tumor volume reached 50 ∼ 100 mm^3^. Then each group was intravenously administered with Saline, NPPA-PTX NPs (12 mg kg^−1^, q3d × 4) and NPPA-PTX NPs (12 mg kg^−1^, q3d × 4) combined with aPD-L1 (24 h postinjection, i.p. 100 μg per mouse) at day 1, respectively. Average tumor growth curves of each treatment group in 4T1 (a) or CT26 (b) tumor-bearing mice. (*n* = 5, mean ± SEM was shown, **p < .01, ***p < .001 compared to the saline group; ^##^*p* < .01 compared to the NPPA-PTX NPs 12 mg kg^−1^ group). Curves showing the body weight change of mice during various treatments in 4T1 (c) or CT26 (d) tumor model. Tumor photograph from 4T1 (e) or CT26 (f) tumor-bearing mice after treatment for 15 days. H&E staining of tumor tissues in 4T1 (g) or CT26 (h) tumor-bearing mice.

The antitumor activity of NPPA-PTX NPs combined with aPD-L1 was evaluated in 4T1 and CT26 tumor-bearing mice. As shown in Figure 6(a,b), the tumor growth was significantly inhibited in NPPA-PTX NPs and a combination of NPPA-PTX NPs/aPD-L1 treatment, compared with the physiological saline treatment group (*p* < .01; *p* < .001, respectively) in 4T1 or CT26 tumor-bearing mice. Especially, in 4T1 or CT26 tumor-bearing mice, compared with NPPA-PTX NPs alone, and combination NPPA-PTX NPs/aPD-L1 treatment significantly improved the antitumor effects (*p* < .01). In 4T1 tumor-bearing mice, the average tumor size at day 15 in NPPA-PTX NPs and combination NPPA-PTX NPs/aPD-L1 treatment groups were 531 ± 124 mm^3^ and 276 ± 111 mm^3^, respectively, compared with 1162 ± 283 mm^3^ in the physiological saline group (*p* < .01; *p* < .001, respectively). The corresponding tumor growth inhibition in NPPA-PTX NPs and combination NPPA-PTX NPs/aPD-L1 treatment groups were about 54.3% and 76.2%, respectively. In CT26 tumor-bearing mice, the average tumor size at day 15 in NPPA-PTX NPs and combination NPPA-PTX NPs/aPD-L1 treatment groups were 872 ± 110 mm^3^ and 538 ± 167 mm^3^, respectively, compared with 1908 ± 484 mm^3^ in the physiological saline treatment group (*p* < .01; *p* < .001, respectively). The corresponding tumor growth inhibition in NPPA-PTX NPs and combination NPPA-PTX NPs/aPD-L1 treatment groups were about 51.9% and 71.8%, respectively. The body weight had no significant changes for each group during the treatment in 4T1 or CT26 tumor-bearing mice (Figure 6(c,d)). In addition, the photographs of isolated tumors at the end of the experiment are shown in Figure 6(e,f). The H&E staining of tumor tissues on 4T1 and CT26 tumor-bearing mice showed that compared with the physiological saline treatment group, NPPA-PTX NPs and combined of NPPA-PTX NPs with aPD-L1 treatment groups both induced necrosis among the tissues (Figure 6(g,h)), which corresponds to the *in vivo* antitumor results shown above.

### 3.7. Immune response of NPPA-PTX NPs combined with aPD-L1 *in vivo*

The strong antitumor effect of NPPA-PTX NPs is likely due to a synergistic effect of both immunotherapy and chemotherapy. To explore the antitumor immune response in combination therapy, tumor tissues were harvested post-treatments in order to analyze the associated immune signals via histological and ELISA.

**Figure 7.**
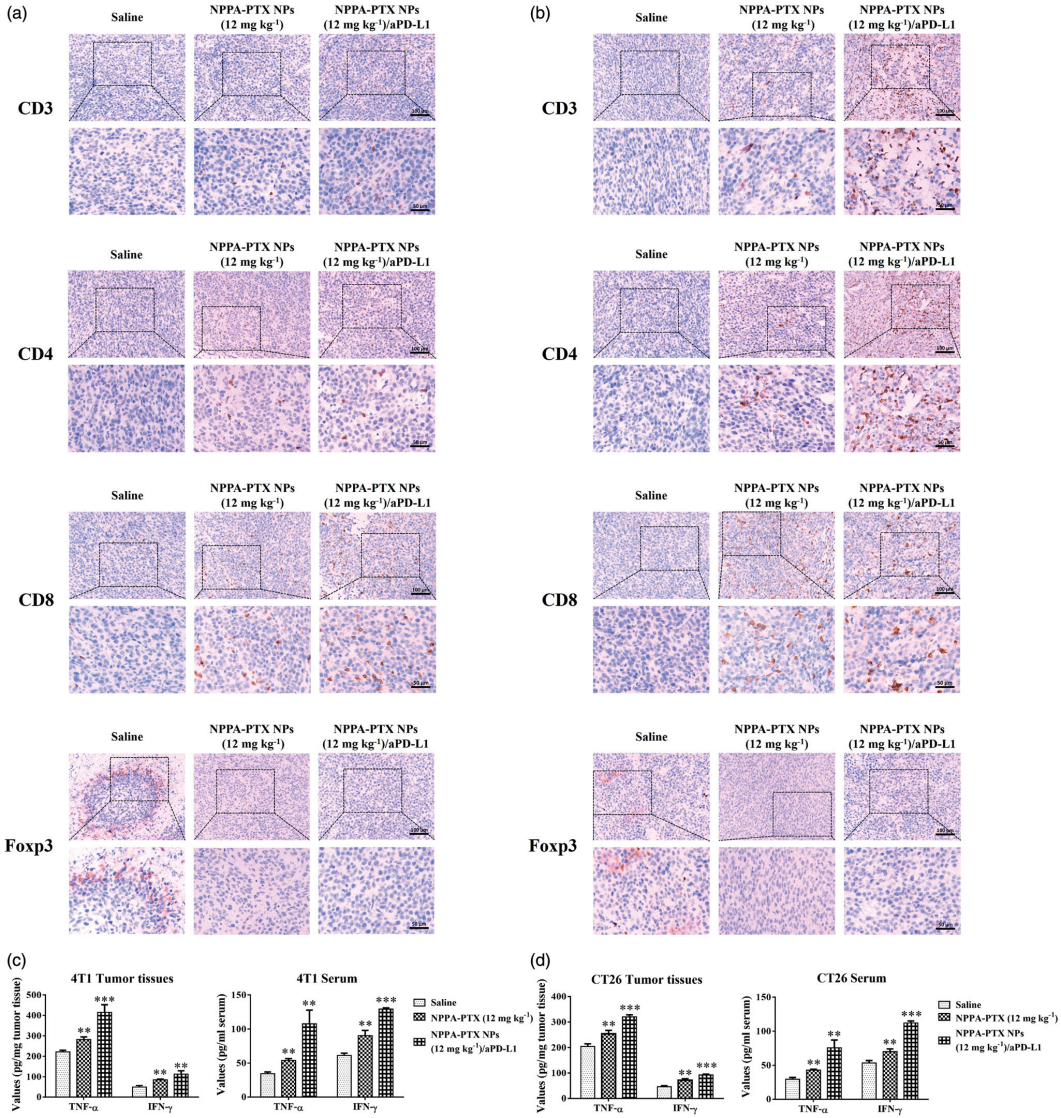
Immune response induced by NPPA-PTX NPs combined with aPD-L1 antibody *in vivo*. Immunohistochemical staining of CD3+, CD4+, CD8+ T cells, and Foxp3+ Tregs in paraffin section of 4T1 (a) or CT26 (b) Tumor tissues (shown in brown). Analysis of expression levels of TNF-α and INF-γ by ELISA assay in tumor tissues and serum of 4T1 (c) or CT26 (d) tumor-bearing mice (*n* = 5, mean ± SEM was shown, **p < .01, ***p < .001).

The tumor infiltration of T cells and the expression levels of PD-L1 in tumor tissues after treatments were measured. In Figure 7(a,b), IHC results showed that chemotherapy using NPPA-PTX NPs and combination NPPA-PTX NPs/aPD-L1 both increased the number of CD3+, CD4+, and CD8+ T cells in tumor tissues, compared to the physiological saline treatment group. On the other hand, the number of Tregs marked by Foxp3+, decreased in NPPA-PTX NPs and combination NPPA-PTX NPs/aPD-L1 groups in contrast to the physiological saline group. Furthermore, the immune cytokines of TNF-α and IFN-γ were further analyzed to evaluate the immunotherapeutic effect. As shown in Figure 7(c,d), significantly increased secretions of TNF-α and IFN-γ were detected in the NPPA-PTX NPs and combination NPPA-PTX NPs/aPD-L1 treatment groups both in serum and tumor tissue in comparison with the physiological saline treatment group. Especially, the combination of NPPA-PTX NPs with aPD-L1 treatment increased the secretions of TNF-α and IFN-γ by 1.87-fold and by 2.34-fold in tumor tissue, by 3.12-fold and by 2.10-fold in serum on 4T1 tumor-bearing mice, respectively. In terms of CT26 tumor-bearing mice, the combination of NPPA-PTX NPs with aPD-L1 treatment increased the secretions of TNF-α and IFN-γ by 1.57-fold and by 2.03-fold in tumor tissue, by 2.56-fold and by 2.09-fold in serum, respectively.

## 4. Discussion

Recent studies have demonstrated that low-dose chemotherapy drugs such as doxorubicin and mitoxantrone not only suppress tumor growth but also elicit antitumor immunity by inducing immunogenic cell death (ICD) of tumor cells (Musetti & Huang, 2018; Zhao et al., 2018). In Figure 3, NPPA-PTX NPs induced the ICD of MDA-MB-231 and 4T1 cells. The essential marker, CRT and HMGB1 were both overexpressed in MDA-MB-231 or 4T1 cell lines, which stimulates the recognition and phagocytosis of antigen-presenting cells (APCs) to dying tumor cells. This process promotes the maturation of DCs, which present the captured antigens on MHC I and MHC II molecules to T cells, resulting in the priming and activation of effector T cell responses against the cancer-specific antigens. The activated effector T cells infiltrate the tumor site and specifically recognize and kill the target cancer cells (Chen & Mellman, 2013; Karasaki et al., 2017). The *in vitro* data showed that NPPA-PTX NPs act as an ICD inducer which stimulated the antitumor response. Moreover, both monotherapy of NPPA-PTX and combination NPPA-PTX NPs/aPD-L1 therapy induced the immune response among tumor tissues (Figure 7(a,b)). In comparison, the combination NPPA-PTX NPs/aPD-L1 treatment resulted in the most remarkable tumor infiltration of CD8+ T cells, which is essential for highly effective tumor immunotherapy (Su et al., 2020). The number of Tregs expressed Foxp3+ decreased in the monotherapy and combined therapy, which confirmed the reversal of immunosuppression (Zhu & Chen, 2019). Current research shows that tumors might escape immune surveillance via PD-1/PD-L1 axis upon NPPA-PTX NPs chemotherapy (Peng et al., 2015; Yang et al., 2020) which highlights the significance of combining the chemotherapeutic agent NPPA-PTX NPs with immune checkpoint blockade molecules like aPD-L1. In addition, it was known that ‘hot’ tumors are T cell-inflamed and highly immunogenic which are usually good responders to immune checkpoint inhibitors (Li & Burgess, 2020). Besides, the higher level of TNF-α and IFN-γ in serum and tumor tissues of combination therapy indicated that NPPA-PTX NPs induced NK cell proliferation, and stimulated TNF-α and IFN-γ production (Jewett et al., 2020). The monotherapy of NPPA-PTX NPs showed lower levels of cytokines than that of combination NPPA-PTX NPs/aPD-L1 treatment, which may be attributed to the inactivation of T cells due to the upregulation of PD-L1. These results demonstrate that combination NPPA-PTX NPs/aPD-L1 treatment stimulated the antitumor immune response to the greatest extent and showed synergistic therapeutic effects over monotherapy with NPPA-PTX NPs.

The administrated dosage is an important factor for the stimulation of antitumor immune response, especially for drugs with cytotoxicity (Sharma & Allison, 2015). It was reported that, even at a low dose (10 mg kg^−1^), nano-PTX could significantly suppress tumor growth more efficiently than immune activation alone (Yang et al., 2020). However, chemotherapy at a high dosage produced indiscriminate cytotoxicity both in tumors and immune cells (DCs, T cells) (Mathios et al., 2016). On the other hand, the non-targeted effect of conventional chemotherapy, immune cell exhaustion, and dysfunction, during treatment is also a problem that needs to be solved. In consideration of the above, in this study, the nano-formulation of PTX NPPA-PTX NPs was proposed as a candidate to elicit antitumor immunity. The characterization of NPPA-PTX NPs (Figure 1) and pharmacokinetics assay suggest that NPPA-PTX NPs are in a stable state during blood circulation after intravenous injection and could specifically distribute to the tumor site via the EPR effect (Lin et al., 2000; Ogawara et al., 2008; Danhier, 2016). The *in vivo* imaging also confirmed the target distribution of NPPA-PTX NPs (Figure S5). These results demonstrate that compared with the traditional PTX formulation, NPPA-PTX NPs decreased immune cytotoxicity as to preserve the antitumor immune responses, which are mediated in the peripheral blood. In other words, NPPA-PTX NPs could protect the immune-activation effects by prodrug-based nano-formulation. The results of the safety evaluation (Figure 5(g–j)) also confirmed this conclusion. Briefly, NPPA-PTX NPs endowed PTX with more effective antitumor activity and better compatibility with the immune system, which makes combination treatment with immunotherapy possible.

According to the pharmacokinetic data of NPPA-PTX NPs, the MRT of released PTX from NPPA-PTX is 4-fold compared to that of PTX from Taxol. The local and sustained drug concentrations among tumor tissues will penetrate the next tumor cell layer resulting in further cell death and possible immune response (Vassileva et al., 2008). The data suggested that NPPA-PTX NPs could stimulate the body to produce sustained anti-tumor immunity.

## 5. Conclusion

In our study, NPPA-PTX NPs act as an ICD inducer that induces the release of DAMPs from tumor cells which can stimulate the recognition and phagocytosis of APCs, promote the maturation of DCs, and enhance the antigen presentation of T cells. This process results in the recruitment and infiltration of CD3+, CDC4+, CD8+ T cells among tumor tissues and decreases the number of Foxp3+ Tregs. The combination of NPPA-PTX NPs/aPD-L1 treatment blocked the PD-1/PD-L1 axis effectively and enhanced the antitumor immune response *in vivo*. In conclusion, NPPA-PTX NPs achieve targeted tumor chemotherapy to improve antitumor activity and stimulate the body to produce strong antitumor immunity, which can be further enhanced by the use of aPD-L1 in combination.

## Supplementary Material

Supplemental Material
