# Supplementary material for: The synergistic antitumor activity of 3-(2-nitrophenyl) propionic acid-paclitaxel nanoparticles (NPPA-PTX NPs) and anti-PD-L1 antibody inducing immunogenic cell death
Source: Drug Deliv. 2021 Apr 19;28(1):800–13. doi: 10.1080/10717544.2021.1909180 (PMC8079060; doi:10.1080/10717544.2021.1909180)
Supplement: Supplemental Material [file IDRD_A_1909180_SM4579.docx]

**Supplementary Material**

**The synergistic antitumor activity of 3-(2-nitrophenyl) propionic acid-paclitaxel nanoparticles (NPPA-PTX NPs) and anti-PD-L1 antibody inducing immunogenic cell death**

**Xiao-Chuan Duan^1,2^, Li-Yuan Peng^3^, Xin Yao^1,2^, Mei-Qi Xu^1,2^, Hui Li^1,2^, Shuai-Qiang Zhang^1,2^, Zhuo-Yue Li^1,2^, Jing-Ru Wang^1,2^, Zhen-Han Feng^1,2^, Guang-Xue Wang^1,2^, Ai Liao^1,2^, Ying Chen^3^, Xuan Zhang^1,2,^***

**^1^**Beijing Key Laboratory of Molecular Pharmaceutics and New Drug Delivery Systems, School of Pharmaceutical Sciences, Peking University, Beijing 100191, People’s Republic of China;

**^2^**Department of Pharmaceutics, School of Pharmaceutical Sciences, Peking University, Beijing 100191, People’s Republic of China;

**^3^**Tianjin Key Laboratory on Technologies Enabling Development Clinical Therapeutics and Diagnostics (Theranostics), School of Pharmacy, Tianjin Medical University, Tianjin 300070, People’s Republic of China

*Correspondence: Xuan Zhang

Department of Pharmaceutics, School of Pharmaceutical Sciences, Peking University, 38 Xueyuan Road, Beijing 100191, People’s Republic of China

Tel/fax +86 10 8280 5765

Email [xuanzhang@bjmu.edu.cn](mailto:xuanzhang@bjmu.edu.cn)


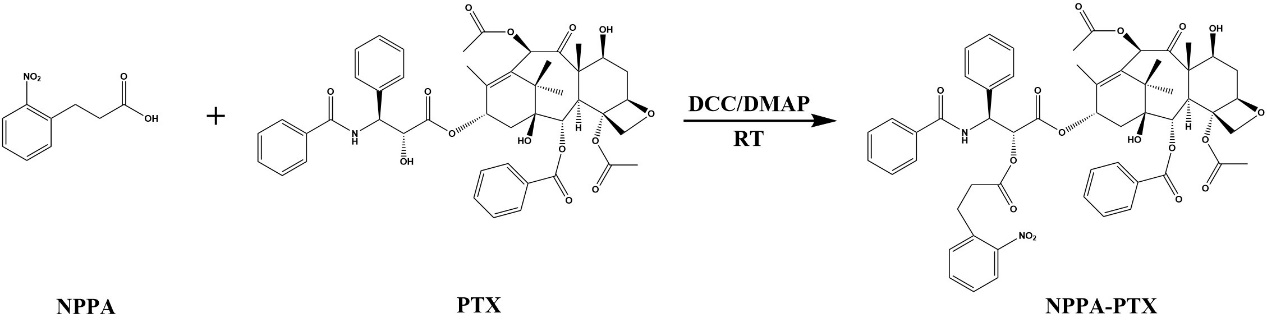


**Scheme 1.** Synthesis of NPPA-PTX


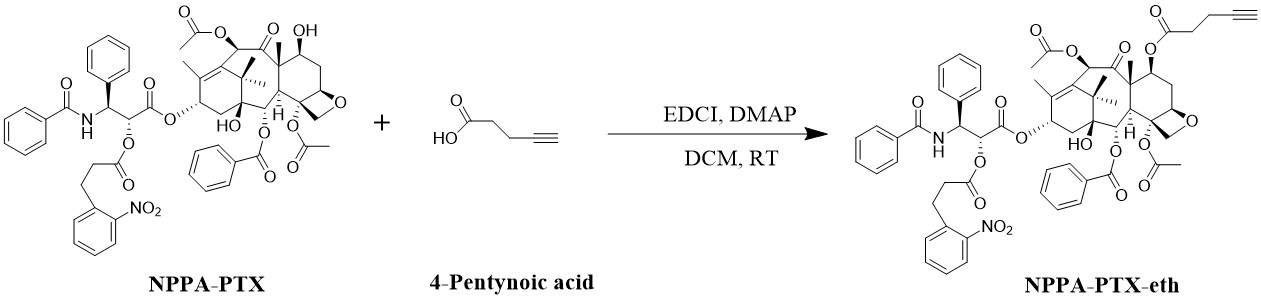


**Scheme 2.** Synthesis of NPPA-PTX-eth


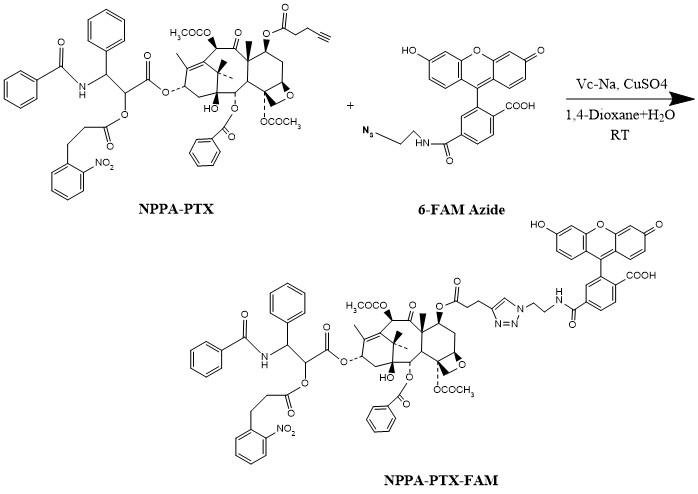


**Scheme 3.** Synthesis of NPPA-PTX-FAM

**
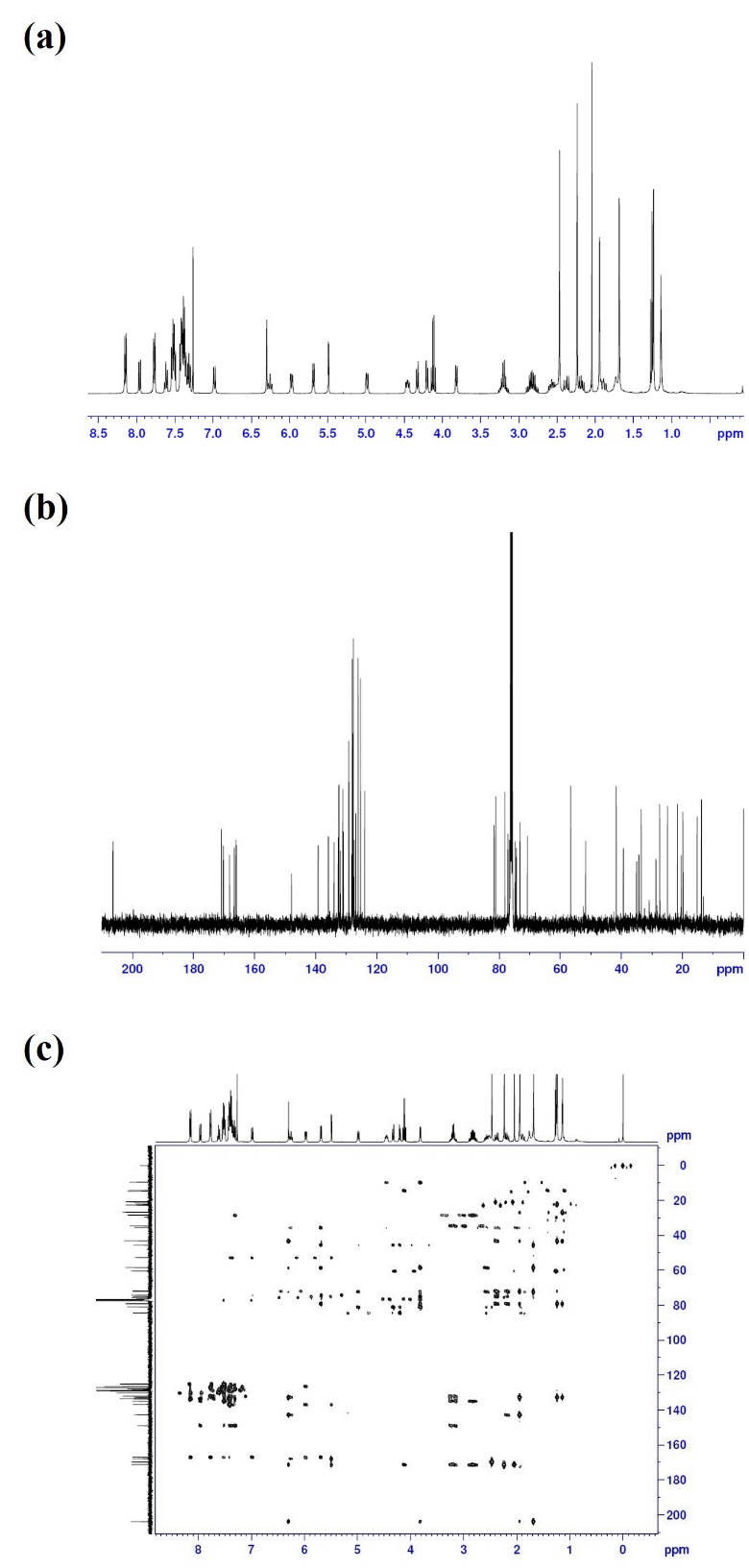
Figure S1.** NMR spectra of NPPA-PTX, ^1^H NMR (a), ^13^C NMR (b) and 1H-13C HMBC (c).


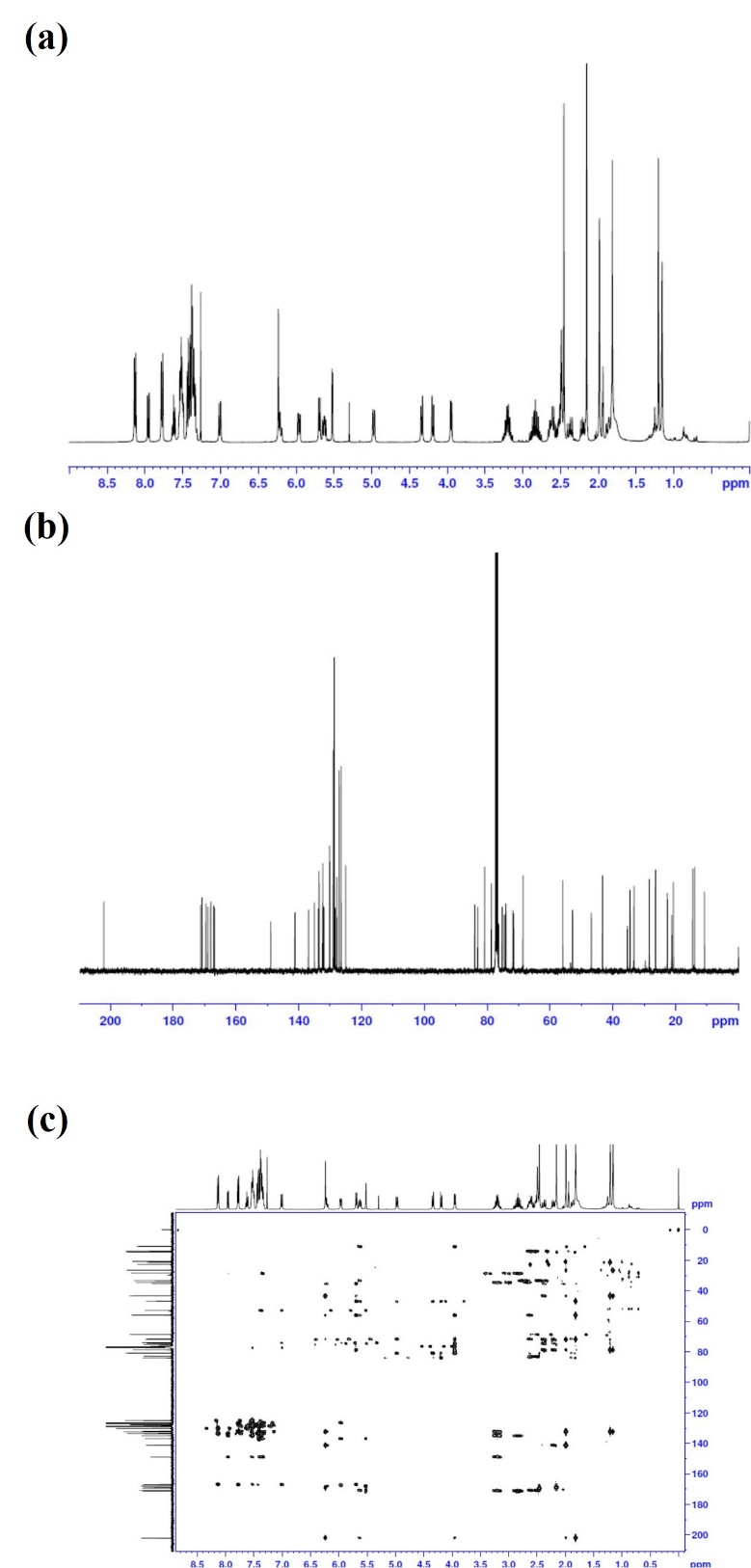


**Figure S2.** NMR spectra of NPPA-PTX-eth, ^1^H NMR (a), ^13^C NMR (b) and 1H-13C HMBC (c).

**
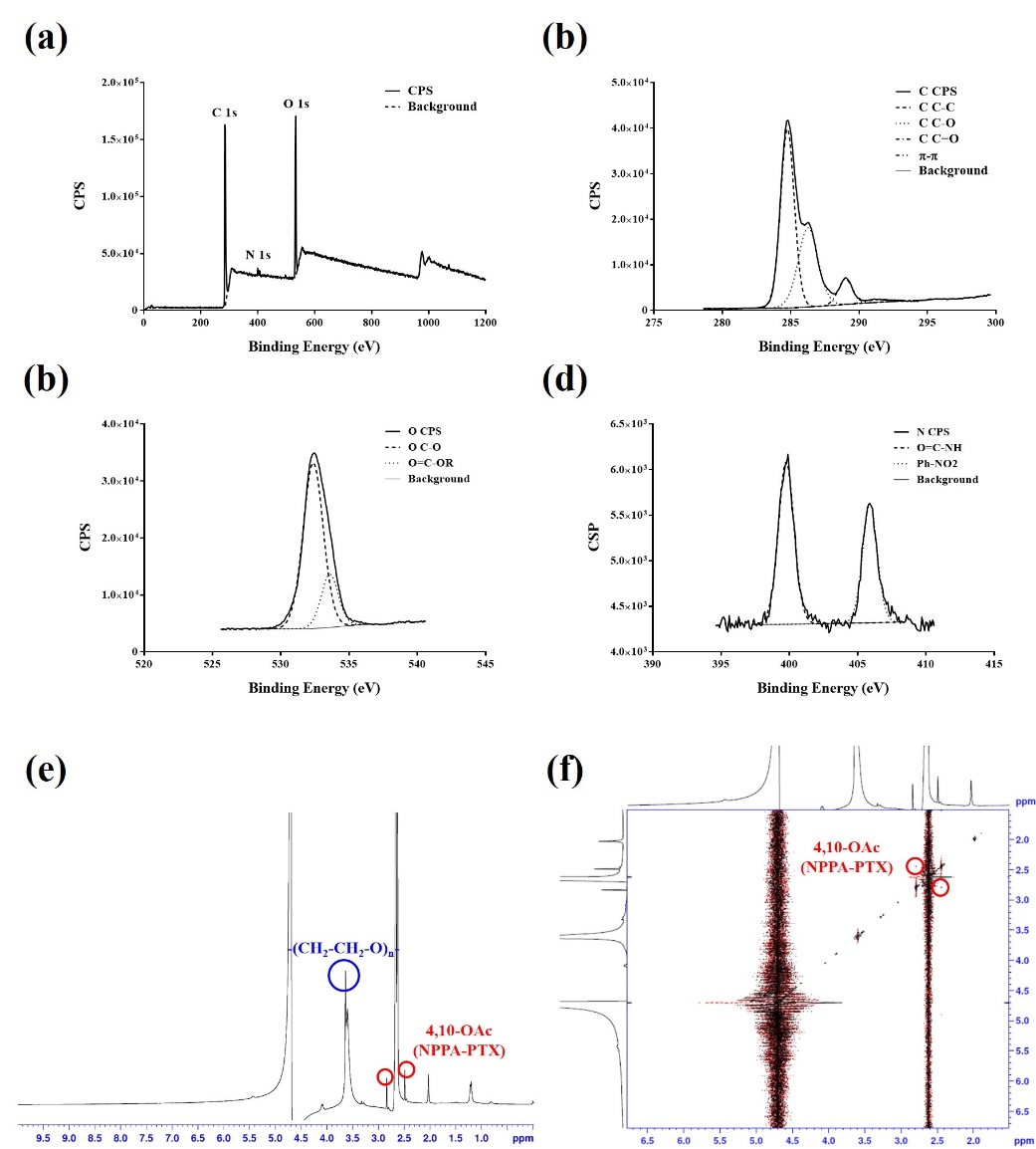
**

**Figure S3.** (a-f) The surface chemical properties characterization of NPPA-PTX NPs. (a-d) X-ray photoelectron spectroscopy was examined on Axis Ultra DLD and (e and f) 1D/2D H-NMR spectrum was examined on AVANCE Ⅲ 400 MHz.

**
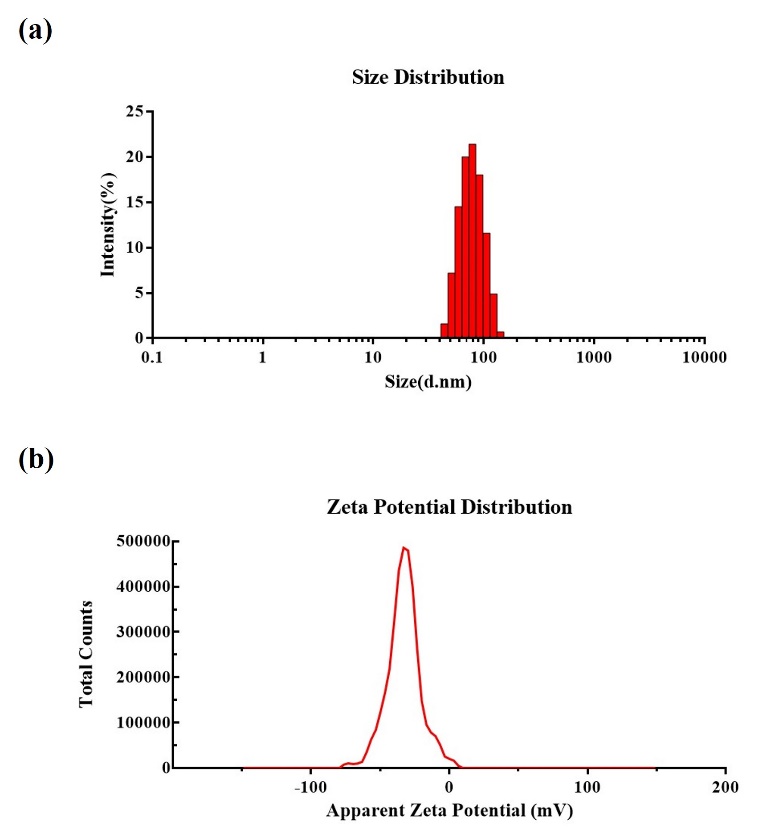
**

**
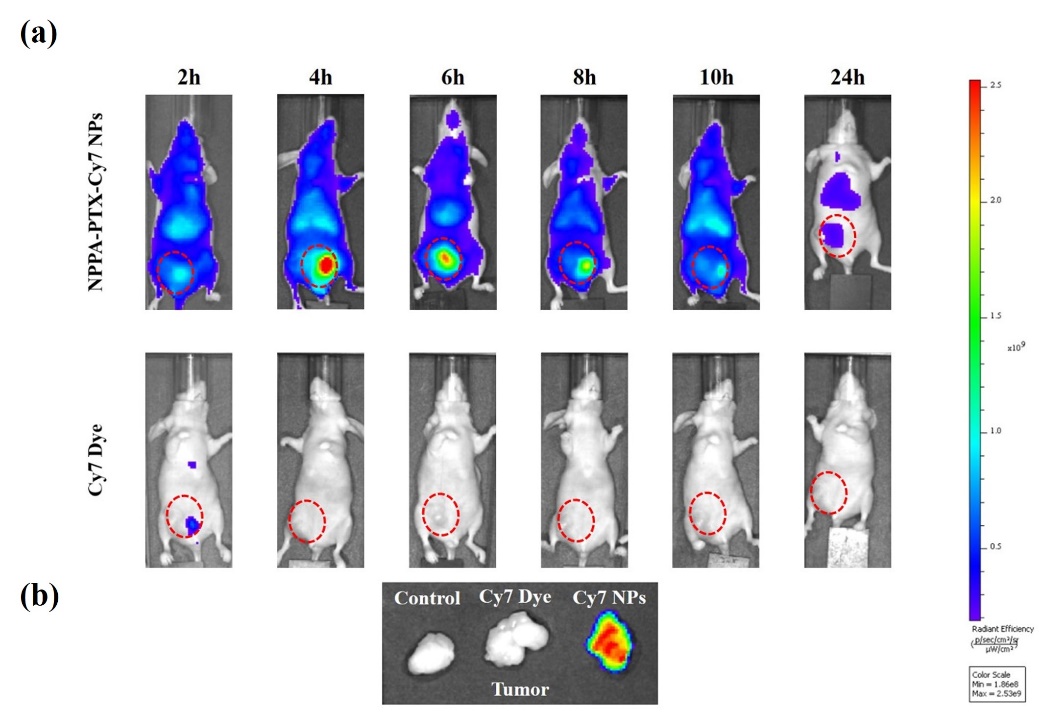
Figure S4.** Particle size (a) and zeta potential (b) of NPPA-PTX-FAM NPs that determined by dynamic light scattering (DLS) measurements on Malvern Zetasizer Nano ZS90 instrument.

**Figure S5.** In vivo imaging of Cy7-labeled NPPA-PTX NPs in HCT116 tumor-bearing nude mice. (a) In vivo whole-body imaging of HCT116 tumor-bearing nude mice after NPPA-PTX-Cy7 NPs administration at 2, 4, 6, 8, 10, and 24 hours, respectively. (b) The ex vivo optical images of tumors of HCT116 tumor-bearing nude mice sacrificed at 24 hours after NPPA-PTX-Cy7 NPs administration.

**Table S1.** Types and concentrations of inhibitors used in the research

| Inhibitors | Function | Concentration (μM) |
| --- | --- | --- |
| EIPA | Inhibitor of macropinocytosis | 20 μM |
| CPZ | Inhibitor of clathrin | 30 μM |
| Hypertonic sucrose | Inhibitor of clathrin | 0.4 M |
| Filipin | Inhibitor of lipid raft | 0.5 μg ml^-1^ |
| MβCD | Inhibitor of lipid raft | 10 mM |

**Table S2.** The concentration of NPPA-PTX in plasma or major organs at 1 h, 4 h, 8 h, 24 h (n = 3, mean ± SEM was shown) and corresponding AUC_0-24h_, AUC tissue/plasma value.

| NPPA-PTX | 1 h  (ng g^-1^ or ng ml^-1^) | 4 h  (ng g^-1^ or ng ml^-1^) | 8 h  (ng g^-1^ or ng ml^-1^) | 24 h  (ng g^-1^ or ng ml^-1^) | AUC_0-24h_ | AUC tissue/plasma |
| --- | --- | --- | --- | --- | --- | --- |
| Plasma/Tissue | AVG | AVG | AVG | AVG |  |  |
| Plasma | 1303.33 ± 251.06 | 159.50 ± 57.23 | 7.05 ± 1.77 | 0.00 | 3235.42 | 1.00 |
| Spleen | 464.00 ± 95.39 | 51.17 ± 23.69 | 2.08 ± 1.80 | 0.00 | 1127.85 | 0.35 |
| Heart | 927.00 ± 152.50 | 72.37 ± 35.60 | 0.00 | 0.00 | 2107.28 | 0.65 |
| Kidney | 1007.33 ± 348.37 | 146.40 ± 109.05 | 3.03 ± 3.27 | 0.00 | 2557.33 | 0.79 |
| Liver | 4076.67 ± 1078.58 | 562.33 ± 264.77 | 9.83 ± 8.52 | 4.97 ± 4.66 | 10259.62 | 3.17 |
| Lung | 743.67 ± 226.43 | 82.53 ± 37.74 | 0.00 | 0.00 | 1776.20 | 0.55 |
| Tumor | 578.00 ± 375.65 | 62.57 ± 43.53 | 8.93 ± 11.42 | 0.00 | 1464.32 | **0.45** |

**Table S3.** The concentration of released PTX from NPPA-PTX in plasma or major organs at 1 h, 4 h, 8 h, 24 h (n = 3, mean ± SEM was shown) and corresponding AUC_0-24h_, AUC tissue/plasma value.

| Released PTX from  NPPA-PTX | 1 h  (ng g^-1^ or ng ml^-1^) | 4 h  (ng g^-1^ or ng ml^-1^) | 8 h  (ng g^-1^ or ng ml^-1^) | 24 h  (ng g^-1^ or ng ml^-1^) | AUC_0-24h_ | AUC tissue/  plasma |
| --- | --- | --- | --- | --- | --- | --- |
| Plasma/Tissue | AVG | AVG | AVG | AVG |  |  |
| Plasma | 1125.00 ± 207.91 | 285.00 ± 109.34 | 25.97 ± 2.07 | 3.81 ± 0.98 | 3537.67 | 1.00 |
| Spleen | 6146.67 ± 90.74 | 4910.00 ± 1290.39 | 547.67 ± 147.78 | 17.90 ± 5.73 | 35098.20 | 9.92 |
| Heart | 4176.67 ± 98.66 | 1553.33 ± 245.42 | 131.33 ± 20.79 | 10.57 ± 3.93 | 15187.87 | 4.29 |
| Kidney | 8573.33 ± 523.86 | 3360.00 ± 1062.40 | 255.67 ± 45.18 | 24.63 ± 4.44 | 31660.40 | 8.95 |
| Liver | 19366.67 ± 2458.32 | 8553.33 ± 3385.05 | 1220.00 ± 30.00 | 84.73 ± 13.57 | 81547.87 | 23.05 |
| Lung | 5386.67 ± 240.07 | 2583.33 ± 482.32 | 377.67 ± 83.19 | 96.43 ± 8.73 | 24363.13 | 6.89 |
| Tumor | 1733.33 ± 30.55 | 1656.67 ± 359.21 | 1380.00 ± 202.24 | 1017.33 ± 71.84 | 31203.67 | **8.82** |

**Table S4.** The concentration of PTX from Taxol in plasma or major organs at 1 h, 4 h, 8 h, 24 h (n = 3, mean ± SEM was shown) and corresponding AUC_0-24h_, AUC tissue/plasma value.

| PTX from Taxol | 1 h  (ng g^-1^ or ng ml^-1^) | 4 h  (ng g^-1^ or ng ml^-1^) | 8 h  (ng g^-1^ or ng ml^-1^) | 24 h  (ng g^-1^ or ng ml^-1^) | AUC_0-24h_ | AUC tissue/  plasma |
| --- | --- | --- | --- | --- | --- | --- |
| Plasma/Tissue | AVG | AVG | AVG | AVG |  |  |
| Plasma | 4930.00 ± 1645.08 | 514.00 ± 475.38 | 31.37 ± 4.90 | 2.53 ± 0.68 | 11992.88 | 1.00 |
| Spleen | 8423.33 ± 76.38 | 4560.00 ± 940.16 | 496.67 ± 140.49 | 28.73 ± 22.28 | 38003.20 | 3.17 |
| Heart | 5603.33 ± 290.06 | 1586.67 ± 546.84 | 172.33 ± 64.83 | 6.16 ± 6.58 | 18532.64 | 1.55 |
| Kidney | 12033.33 ± 513.16 | 3783.33 ± 1751.69 | 293.67 ± 69.51 | 20.37 ± 4.30 | 40407.93 | 3.37 |
| Liver | 34800.00 ± 953.94 | 9783.33 ± 5121.60 | 1457.67 ± 463.90 | 74.17 ± 14.02 | 119011.67 | 9.92 |
| Lung | 7920.00 ± 1037.64 | 2690.00 ± 1330.11 | 461.3 3± 55.63 | 112.67 ± 11.55 | 30769.67 | 2.57 |
| Tumor | 2463.33 ± 115.90 | 1969.00 ± 931.47 | 1313.33 ± 119.30 | 922.67 ± 186.18 | 32332.83 | **2.70** |

**Table S5.** The IC_50_ values (μM) of PTX and NPPA-PTX NPs in MDAMB-231 cells or HCT116 cells (n = 3, mean ± SD was shown).

|  | PTX (μM) | NPPA-PTX NPs (μM) |
| --- | --- | --- |
| MDA-MB-231 | 0.063 ± 0.0033 | 0.072 ± 0.0045* |
| HCT116 | 0.30 ± 0.02 | 0.23 ± 0.02* |

*P < 0.05, compared with PTX.
